# Nutrition profiles of farm households across different farming systems in Ethiopia: Unpacking the determinants and implications for nutrition‐sensitive interventions

**DOI:** 10.1002/fsn3.4194

**Published:** 2024-04-28

**Authors:** Beruk Berhanu Desalegn, Birhanu Biazin, Tilahun Amede, Jan Low

**Affiliations:** ^1^ College of Agriculture, Hawassa University Hawassa Ethiopia; ^2^ International Potato Center Tamale Ghana; ^3^ International Crops Research Institute for the Semi‐Arid Tropics (ICRISAT) Addis Ababa Ethiopia; ^4^ Alliance for Green Revolution in Agrica Addis Ababa Ethiopia; ^5^ International Potato Center Nairobi Kenya

**Keywords:** farming systems, irrigation, nutrition profiles, nutrition‐sensitive, root crops, vitamin, wild coffee

## Abstract

Tackling nutrition insecurity remains a critical challenge in developing countries. In the predominantly rain‐fed and smallholder‐based farming systems of Ethiopia, production diversity and livelihood strategies of the farm households vary across geographic areas. However, the effects of household socioeconomic characteristics, production diversity, and household incomes on nutrition profiles in distinct settings have been inadequately understood. Therefore, this study was undertaken to examine the association of farming system type, sources of income, and household wealth status with household nutrition profiles in three remote locations such as Mennisa, Welmel Tiqa, and Agam Wuha that represent root crops‐based farming, maize‐based semi‐pastoral farming, and teff‐based cereal farming systems, respectively. A combination of qualitative and quantitative data collection techniques was employed. A multistage sampling procedure was used to select a total of 265 smallholder households for the structured survey interviews. Standard statistical tests and Tobit regression analyses were performed after determining the wealth category of each household. Results revealed a diversity of income sources used by each of the farm households with average values of 9 in Mennisa, 10 in Agam Wuha, and 11 in Welmel Tiqa, with the contributions of each income source varying by household wealth category and location. As expected, expenditures on food significantly exceeded those on non‐food categories for poor households and vice versa for rich wealth households. The average total food variety score (FVS) for Welmel Tiqa was twice that for Agam Wuha, confirming the need for site‐specific nutrition profile assessments. Despite the observed differences in household nutrition profiles among wealth categories and locations, the apparent intakes of vitamin A, vitamin B_12_, vitamin D, and calcium were consistently below the population‐level estimated average requirements across all locations. The number of adequately consumed nutrients by farm households was negatively associated with family size, age of household heads, livestock holdings, wealth categories, and irrigation use, and positively associated with crop production diversity, income diversity, and FVS. The negative association between irrigation use and nutrition security was likely due to the focus on producing crops with a high market value on land under irrigation, coupled with ineffective allocation of generated income for enhancing household nutritional outcomes. Therefore, programs that include irrigated agriculture investments should consider adopting a more integrated nutrition‐sensitive interventions, including consideration of locally adapted nutritious crops, such as orange‐flesh sweet potato, to address critical deficiency of Vitamin A, nutrition training coupled with development of recipes and cooking demonstrations, and marketing and promotion for nutritious crops.

## Graphical abstract

The objective of the study was to examine the association of farming system type, sources of income, and household wealth status with household nutrition profiles in three remote locations such as Mennisa, Welmel Tiqa, and Agam Wuha that represent root crops‐based farming, maize‐based semi‐pastoral farming, and teff‐based cereal farming systems, respectively. Despite there being differences in household nutrition profiles among wealth categories and locations, the apparent intakes of vitamin A, vitamin B12, vitamin D, and calcium were consistently below the population level Estimated Average Requirements (EARs) across all locations. The number of adequately consumed nutrients by farm households was negatively associated with family size, age of household heads, livestock holdings, wealth categories, and irrigation use, and positively associated with crop production diversity, income diversity, and food variety score. The negative association between irrigation use and nutrition security was likely due to the focus on producing crops with a high market value on land under irrigation, coupled with ineffective allocation of generated income for enhancing household nutritional outcomes.

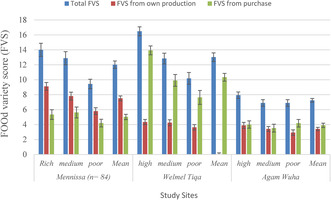

## 1 INTRODUCTION

Undernutrition and micronutrient deficiencies remain critical challenges in many developing countries where the most affected are smallholder farmers (Kramer & Allen, 2015; Sibhatu & Qaim, 2018a, 2018b). They are identified as serious global public health problems and recognized as major underlying causes for maternal and child morbidity, mortality, and disability‐adjusted life years (UNICEF, 1998; Black et al., 2008; Caulfield et al., 2004; Desalegn et al., 2018; Müller & Krawinkel, 2005; Stevens et al., 2015). These problems are particularly severe in sub‐Saharan Africa (SSA) countries where smallholder‐based agriculture is the mainstay of rural livelihood (IFPRI, 2017; Sibhatu & Qaim, 2018a, 2018b). As a result, nutritional deficiencies are still among the major causes of premature deaths, disease, physical and mental growth retardation, and other types of health problems in this region (IFPRI, 2017; Prendergast, 2014). Like other SSA countries, undernutrition and micronutrient deficiency are the major public health problems in Ethiopia (Aguilar et al., 2014; Gebremedhin et al., 2013). Half of the country's rural inhabitants chronically live “below the food poverty level of 2,200 K/calorie equivalent per adult per day” (Aguilar et al., 2014). Different studies in Ethiopia witnessed Vitamin A among the major public health problems in the country (Demissie et al., 2010; Kidane et al., 2013; Eyeberu et al., 2023; Fite et al., 2023; Herrador et al., 2014; Sahile et al., 2020).

Inadequate production of food being considered as the main cause of food and nutrition insecurity in Africa, substantial efforts and commitments have been made to boost agricultural production (Sasson, 2012). The major efforts of boosting agriculture in Africa have focused on the use of fertilizers, better seeds, irrigation investments, extension services, and improved market access (Jama & Pizzaro, 2008; You et al., 2011). Crop choice by smallholders in Africa is influenced by local food habits, markets, traditions, and government policy (Tittonell et al., 2010), with hunger and income generation considerations typically dominating over diet quality. However, during the past decade there have been increasing calls for greater emphasis on nutrition‐sensitive agriculture, as there has been increased awareness of the significant economic loss and health burdens caused by high levels of malnutrition. The United Nations' 2030 Agenda for Sustainable Development (United Nations, 2017) and the integration of nutrition interventions into the Comprehensive Africa Agriculture Development Program investment plans (Rampa & van Seters, 2013) are two examples. Furthermore, the new emerging global consensus in 2020 revealed that the efforts to end both hunger and all forms of malnutrition require the transformation of Food Systems at global level, in order to build a more resilient, sustainable system that can assure access to healthier and affordable diets for all (FAO, IFAD, UNICEF, WFP & WHO, 2020).

For the successful implementations of these initiatives, it is crucial to understand the interplay between production diversity and food diversity on farm household nutrition security in developing countries (Workicho et al., 2016; Qaim & Sibhatu, 2017; Huluka & Wondimagegnhu, 2019). Beyond correlating production and food diversity to farm household nutrition, there needs to be a critical look into the role of the various production systems and livelihood strategies on overall income and household food utilization. For instance, the production diversity of smallholder farmers might be high in mixed perennials (e.g., coffee and other fruits) and annual cropping systems, such as home‐garden agroforestry (Negash et al., 2012), or mixed cereals and root crops farming systems. The question is whether farm households are consuming only few of their more nutritious food crops while selling the majority. Moreover, those households that rely heavily on commercial crops such as coffee depend on purchased foods to feed their families. The effect of wealth status and socioeconomic inequalities on household nutrition profiles has been less studied for farm households in dryland areas (Van de Poel, 2008).

Therefore, the objective of this study was to examine the interplay between farming systems, socioeconomic characteristics, and farm household nutrition profiles at three remotely located dryland sites in Ethiopia and determine whether a level of adequacy was being reached for energy, protein, and 14 micronutrients among different wealth categories. The farming systems represented were: (1) Wild coffee mixed with maize and livestock farming system in southeastern Ethiopia around Welmel Tiqa area of Oromia region, (2) mixed root crops and cereal‐based farming system in southwestern Ethiopia around Mennisa area of Wolayta, and (3) teff‐based cereal and legumes mixed farming system in northwestern Ethiopia around Agam Wuha area of Amhara region. This study was linked to the Participatory Small‐Scale Irrigation Development Program (PASIDP) of Ethiopia, which has been funded by the International Fund for Agricultural Development (IFAD), to develop more than 120 community‐managed small‐scale irrigation schemes with the aim of enhancing both farm incomes and household nutrition. Hence, this baseline study was used to assess the farming households' current nutrition profile, thus enabling the identification of suitable crops for nutrition‐sensitive irrigated agriculture development.

## 2 MATERIALS AND METHODS

### 2.1 Description of the study areas

**FIGURE 1:**
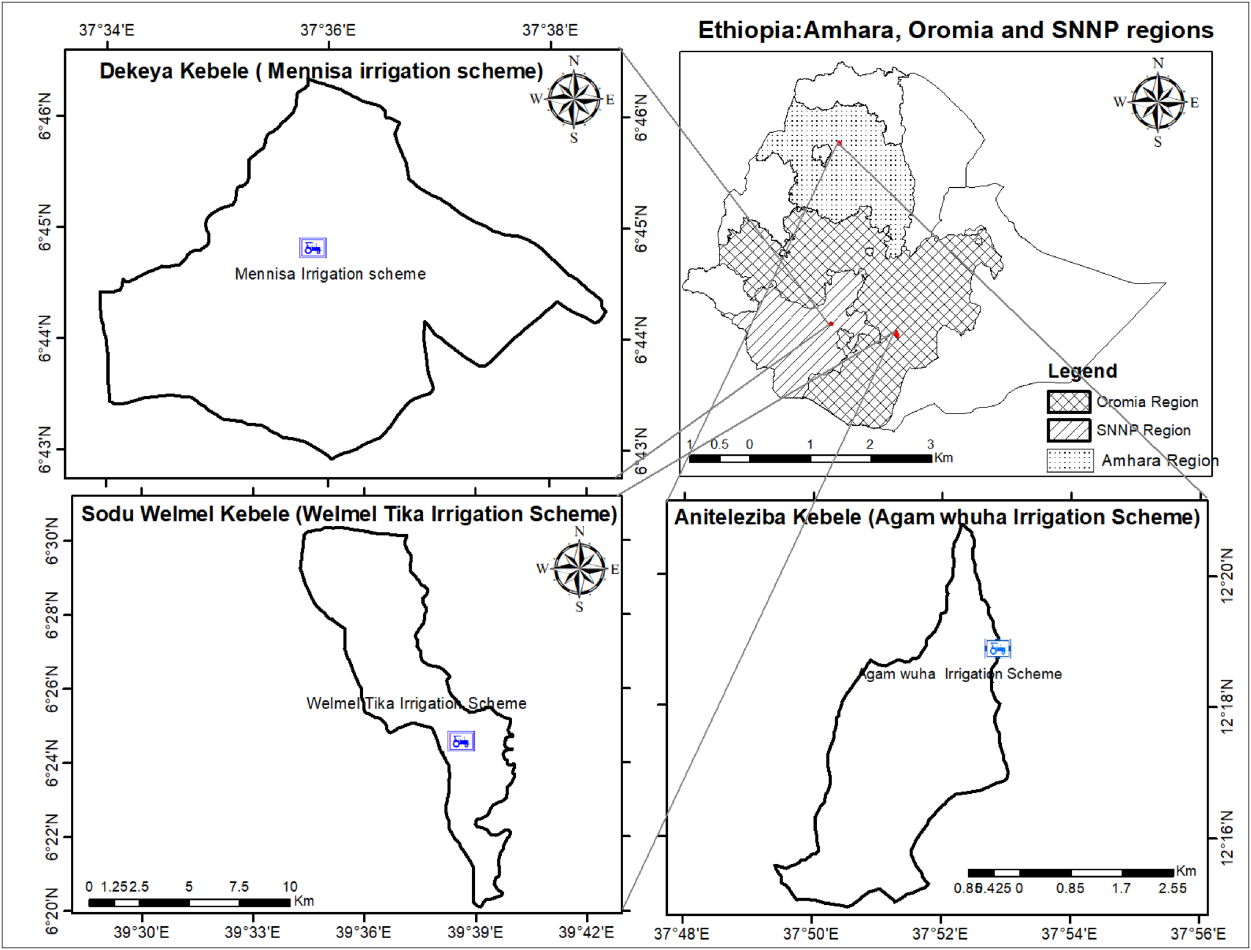
Location map of the study areas.

**FIGURE 2:**
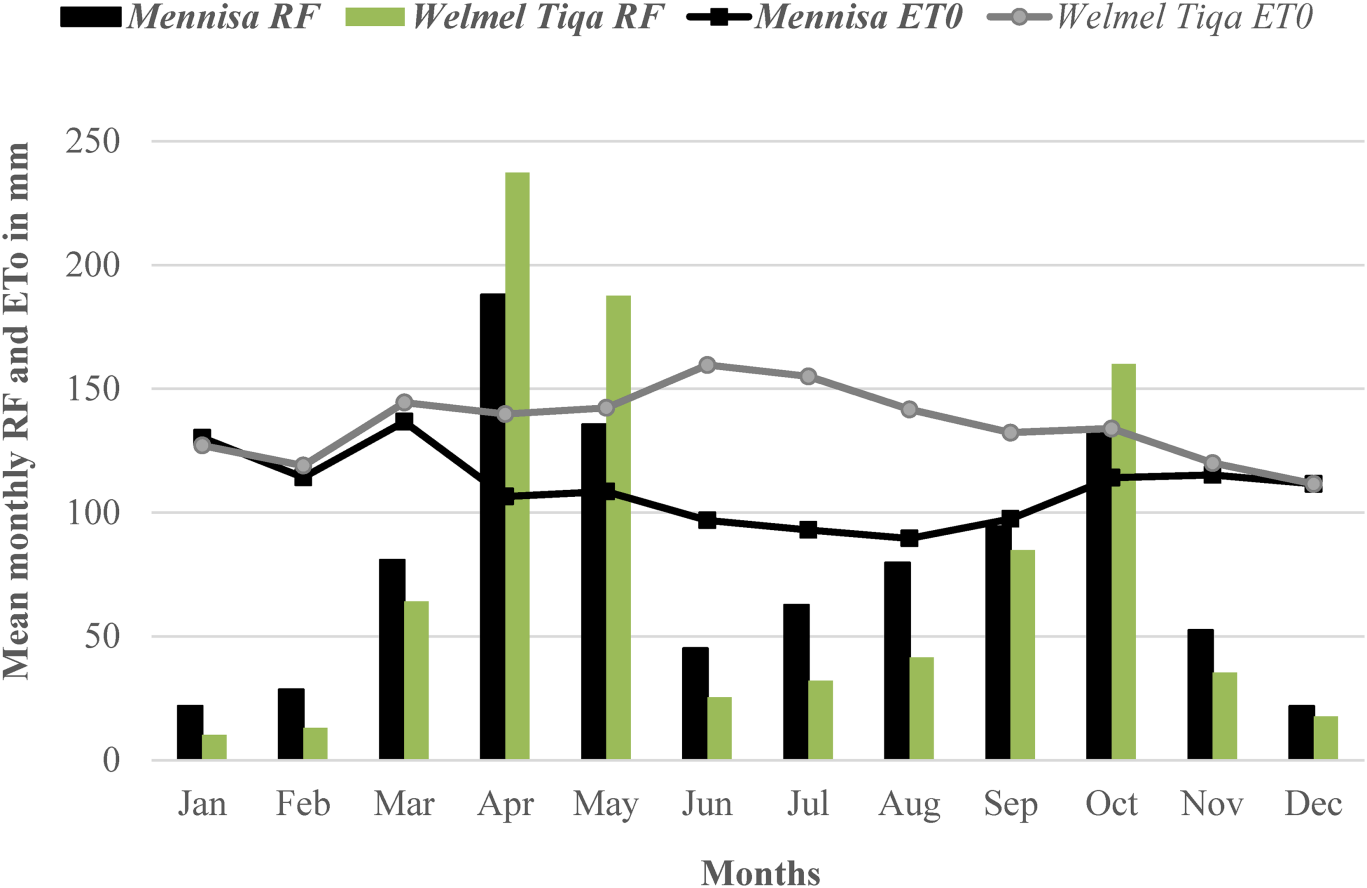
Mean monthly precipitation and reference evapotranspiration (ETo) based on 38 years of data (1981–2018) of Mennisa and Welmel Tiqa study sites in Ethiopia. Data were not available for the Agam Wuha site.

The geographic locations of the three studies areas are depicted in Figure 1, showing Welmel Tiqa in the Harena Buliq district of Southeastern Oromia region, Mennisa in the Offa district of Wolayita in southern Ethiopia, and Agam Wuha in the West Belessa district of the Northern Amhara region. The mean altitudes of Welmel Tiqa, Mennisa, and Agam Wuha areas were 1070, 1670, and 1710 m, respectively. Two study sites (Welmel Tiqa and Mennisa) have bimodal rainfall patterns, the short rains (locally known as *Belg*) from March through May and the long rains (locally known as *Meher*) from June/July through October (Figure 2). However, there is only one main rainy season (June through September) in Agam Wuha (data not available). The gap between the mean annual precipitation and reference evapotranspiration (ETo) is higher in Welmel Tiqa than in Mennisa, implying higher crop water stress in the former. Root crops, such as cassava (*Manihot esculenta*), sweet potato (*Ipomoea batatas*), and enset (*Ensete ventricosum*) are dominant crops in Mennisa, while haricot beans (*Phaseolus vulgaris*), maize (*Zea mays*), and fruits (coffee, mango, avocado, and banana) are also important. Maize and haricot beans are the dominant crops in Welmel Tiqa, while the stimulant khat (*Cata edulis*) is an expanding cash crop. Wild coffee, subsistence crop farming, and livestock are important sources of livelihood in Welmel Tiqa, where the local communities have been shifting from pastoral to semi‐pastoral farming systems over the last 40 years. Root crop production followed by livestock sales and casual labor participation are important means of income in Mennisa. These sites represented three of the most important farming systems in Ethiopia (Amede et al., 2017).

### 2.2 Methods

#### 2.2.1 Sampling technique

A multistage sampling technique was employed in this study. First, three different administrative regions, Amhara, Oromia, and Southern Nations, Nationalities, and Peoples' (SNNP) regions, have been purposively selected to represent remotely located distinct farming and agro‐food systems. A representative district was selected from each of the three regions. Hence, West Belessa district in Amhara region, Harena Buliq district in Oromia region, and Offa district in SNNP region were purposively selected as community‐managed irrigation schemes have been recently initiated and the future investments in nutrition‐sensitive irrigated agriculture development were to be made. The specific study sites where the detailed study was undertaken encompass Agam Wuha in West Belessa, Welmel Tiqa in Harena Buliq, and Mennisa in Offa districts. Farm households have been categorized into three wealth categories (rich, medium, and poor) based on locally perceived wealth criteria, hypothesizing that wealth status significantly affects the agricultural production and nutrition profiles of farm households. Hence, 8–10 key informants who encompassed extension agents, local administrators, and knowledgeable farmers from different geographic locations in the study sites categorized all local farm households as poor, medium, and rich based on their local criteria, such as the number of corrugated metal roofs, land size, annual income, capacity of financing for schooling, cattle number, fixed assets (e.g., coffee, eucalyptus tree, and mango tree), length of the own production ability to feed household member, and meat consumption pattern. Once the number of households in each wealth category was identified, sample respondents were randomly selected from each category for detailed interviews. A total of 282 households were expected to have participated from the three districts (84 from Agam Wuha, 90 from Mennisa, and 108 from Welmel Tiqa); however, 265 households were included in this study (94% response rate), of which 31.32% (83 households) participated from Agam Wuha, 31.70% (84 households) from Mennisa, and 36.98% (98 households) from Welmel Tiqa. These encompass 88 rich, 90 medium, and 87 poor wealth categories across the three study sites. The variations in the sample sizes among the three sites were due to differences in the total households and the limited number of rich households in specific locality.

#### 2.2.2 Data collection

A combination of qualitative and quantitative data collection techniques was employed. Qualitative data were collected through key informant interviews, focus group discussions (FGDs), and field observations to get preliminary information about the agriculture and food systems, and the wealth criteria. There were 8–10 key informants who included district and kebele (the lowest administrative unit in Ethiopia) level agriculture office heads, frontline extension staff members, head of the kebele administration, and elderly farmers judged to have long‐term knowledge about the area. After key informants helped us to categorize the farm households into rich, medium, and poor, three FGDs were undertaken, comprising of one discussion per wealth category, in each of the three study sites. Each FGD engaged 6–8 discussants, comprising up to two women in a given wealth category. An experienced facilitator posed key questions, including major crops grown during the different cropping seasons, major means of livelihoods and household incomes, major food systems and interplay between crop production and consumption, proportion of each crop yields consumed and sold; food security status throughout the different seasons, and major recipes prepared in the study area.

Quantitative data were gathered using a structured household survey. The questionnaire was developed in English and translated into each of the three local languages: Amharic in Agam Wuha, Afaan Oromo in Welmel Tiqa, and Wolayitegna in Offa districts, respectively. The survey questionnaire was slightly modified following the results from the key informants and FGDs. The key issues addressed in the survey encompassed sociodemographic and economic characteristics, major livelihood systems, cropping patterns (major crop types, productivity, and changing trends), food consumption for the household assessed as per the consumption of food items in the last 7 days prior to the survey using recall method, and interplay between agriculture and food systems. The food consumption section of the questionnaire included the list of food items, the total amount of food items consumed for the last 7 days at the household level, proportion of food consumption from purchase and own production, and also the type of recipes that are produced from specific food items. The interviews were undertaken with both the husband and the wife of a given household. In the male‐headed households, the men were active in responding to questions related to household characteristics, cropping patterns, and major means of livelihood, while the women responded to questions mainly related to food consumption levels and types and recipes prepared, due to the mothers being responsible to prepare family foods and provide them to the family members; while the males are responsible for the agricultural practices and household incomes in the farm households in Ethiopian context. Whereas as in the female‐headed households, the woman responded to all the questions, as all the activities are led by the mothers. However, in both cases, whenever relevant, the mothers were asked to collect data in male‐headed households. These measures helped us to collect appropriate and accurate data to meet the intended objective of the study.

The cropping patterns and yield estimates of the previous short rainy season (Belg) and long rainy season (Meher) were undertaken using recall techniques. The types of crops grown on each land parcel, area coverage, and yields were obtained. In cases where perennials like banana or coffee were intercropped with shorter duration food crops, the area coverage and annual yields were estimated. The crops were then ranked from the highest to the lowest by season, based on season‐specific area coverage and yield. Most farm households had off‐farm income sources as well; in total, more than 10 different livelihood strategies were identified. Subsequently, the annual incomes from each of the different income categories were estimated and then combined to estimate the total household income. When different members of the household had income sources, the information obtained was triangulated by asking both the husband and wife.

Data on household food consumption during the last 7 days were collected by adapting section 5 food list of the Ethiopian socioeconomic survey conducted in 2015/2016 (Central Statistical Agency [CSA], 2016). Given that mothers are mainly responsible for selecting which foods are to be prepared, they were asked about whether they consumed each food on the list of 35 food items in the questionnaire and if so, asked to estimate the quantity consumed by the household members during the past 7 days. For some cases such as oil and salt, the mothers could estimate only the amounts consumed per month, and the enumerator adjusted the value to the equivalent over 7 days. If a specific food item eaten by the household was not found on the list, then this item was added to the questionnaire and the amount consumed obtained by recall. Whether the food item was own produced or purchased and the type of food recipes usually prepared from the specific food items were recorded. The types of recipes were identified from the list of commonly prepared recipe list and the households were also asked if additional recipes were prepared in their households. The 7‐day recall household level consumption data have been used by many studies to calculate food security at household level using energy intake as a cutoff point (Mekonen et al., 2021, 2023; Tefera & Tefera, 2014). However, in this study, we used it to determine the nutrition profile of households, which is detailed in the Data analyses section of this study.

#### 2.2.3 Data analyses

Statistical analyses using Statistical Package for Social Sciences (SPSS) window version 25 (IBM Corporation, Armonk, NY, USA) were undertaken, including descriptive statistics, analyses of variance (ANOVAs), the Kruskal–Wallis test, chi‐squared tests, and non‐linear regression analyses. Prior to the analyses, data were cleaned and organized properly. The overall adult male equivalent value for each household was calculated considering the age and sex distributions of the household members following the protocol used in Ethiopian Socioeconomic Survey (CSA, 2012). Likewise, the tropical livestock unit (TLU) for an individual household was obtained by summing the TLU values, which was calculated by multiplying the assigned TLU for the specific livestock type with the number of each specific livestock type (Tibebu, 2007).

The total amount of each food item consumed at the household level during the last 7 days preceding the survey was converted into the edible portion by subtracting the refuse proportion for the specific food items using figures provided in the Ethiopian food composition tables (Ethiopian Nutrition Institute [ENI], 1981; Ethiopian Health and Nutrition Research Institute [EHNRI], 1998). Amounts recorded in local measuring utensils were converted into standard units (g or kg). Then, using the Ethiopian food composition tables (EHNRI, 1998; ENI, 1981), we constructed nutrient profiles using the food items consumed in the households in the study sites (EHNRI, 1998; ENI, 1981). The missing nutrients were adapted from the United States of America Department of Agriculture (USDA) food composition database considering the moisture content for each specific food item (United States Department of Agriculture (USDA), 2018). The nutrient profile prepared for each of the food items was entered in the NutriSurvey (NS) software per 100 g basis. Then, the total amount of each food item consumed (g) in the specific household in the 7 days preceding the survey was entered into the NS and averaged to estimate the average daily consumption per household. Once this was completed, the result was exported to SPSS, and the estimated apparent energy and nutrient intakes at the household level were divided by the adult total male equivalent value calculated for the specific household. Then, the apparent intakes of energy and other 15 nutrients constructed by this study were compared against the estimated average requirements (EARs) for an adult male (30 < *X* ≤60 years) to identify households at high risk of inadequate intake of an individual nutrient, based on being less than the EAR cutoff; otherwise, it was classified as adequate (Baye et al., 2019; Bermudez et al., 2012; CSA, 2012; Institute of Medicine [IOM], 2001, 2005, 2006, 2011). Accordingly, it was possible to count the number of apparently adequate nutrient intakes for each sample household for the 15 nutrients (excluding energy) considered in this study.

The Food Variety Score (FVS) has been defined as the number of food items eaten during a given period and is calculated by simply counting different food items with each given the same weight (Argaw et al., 2021; Hatløy et al., 1998; Seyoum Keflie et al., 2018). Accordingly, the total FVS for each household was computed by simply counting the number of food items consumed within 7 days prior to the survey day. Similarly, the FVSs sourced from own production or purchased from local markets were computed separately.

Further statistical analyses were conducted after checking for normality of continuous data using the Kolmogorov–Smirnov test, histograms, and box plots. Once the normality of the data of a given variable was confirmed or an appropriate adjustment made, a mean comparison among the different wealth categories for a given study site was determined using one‐way ANOVA. The non‐normally distributed data were analyzed using the non‐parametric Kruskal–Wallis test and the subsequent results presented as medians with interquartile ranges. Furthermore, the categorical data were tested for association using the chi‐squared test.

Finally, the empirical relationship between the number of adequately consumed nutrients in each site and the aggregated data for the three study sites and there potential determinants were examined using a Tobit regression model. The Tobit or censored regression model is designed to estimate linear relationships between variables when there is either left‐ or right‐censoring in the dependent variables. It is a class of regression model in which the observed range of the dependent variables is censored in some way. In this study, the dependent variable (number of nutrients adequately consumed by a given household) is censored at 1 from below and censored from above at 15. Since the dependent variable is restricted within certain values, applying the usual ordinary least squares regression would result in biased and inconsistent parameters, whereas the Tobit censored regression could yield consistent parameter estimates (Amemiya, 1973). More importantly, the Tobit model has an advantage of yielding asymptotically efficient estimates (Heckman, 1979; Wooldridge, 2010).

The Tobit model, which reveals the empirical relationship between nutrition adequacy and determinants, could be specified as Equation (1):

(1) $$\left.\begin{array}{c} N_{i}=\beta_{0}+B_{1}{\left(\text{Landsize}\right)}_{i}+B_{2}{\left(\text{Familysize}\right)}_{i}+B_{3}{\left(\text{Headsex}\right)}_{i}+B_{4}{\left(\text{Headeduc}\right)}_{i}\\ +B_{5}{\left(\text{Livestock}\right)}_{i}+B_{6}{\left(\text{Cropdiverse}\_\text{belg}\right)}_{i}+B_{7}{\left(\text{Cropdiverse}\_\text{meher}\right)}_{i}\\ +B_{8}{\left(\text{Cropdiverse}\_\text{Year}\right)}_{i}+B_{9}{\left(\text{Irrigation}\_\mathrm{Use}\right)}_{i}+B_{10}{\left(\text{Income}\_\text{diversity}\right)}_{i}\\ +B_{11}{\left(\text{Annual}\_\text{Income}\right)}_{i}+B_{12}{\left(\text{Food}\_\text{expenditure}\right)}_{i}+B_{13}{\left(\text{FoodVariety}\right)}_{i}\\ +B_{14}{\left(\text{Wealth}\_\text{Status}\right)}_{i}+U_{i} \end{array}\right\}\ldots .$$

where *N* = the number of apparently adequately consumed nutrients by a given farm household and it is the dependent variable of the Tobit model [1…15]. *B* = parameters to be estimated, which show the magnitude of change in the dependent variable (*N*) due to a unit change in a given variable (*B*_1_–*B*_14_), controlling for other factors affecting *N*. ***U*** = the error term or residual term, which captures the part of change in *N* not attributed to the included independent variables and also capture measurement and specification errors. It is assumed to follow normal distribution with equal variances for a given *X*:$U_{i}\sim N\left(0,\delta^{2}\right)$. ***i*** = the unit of analysis, household in our case.

Therefore, the Tobit model empirically examined the relationship between the number of apparently adequate nutrients (*N*) and carefully chosen independent variables (*B*_1_‐_14_). The dummy variables encompass sex (0 for female and 1 for male) of the respondent and irrigation use (0 for non‐user and 1 for irrigation user). The categorical variables encompass educational status (1 for illiterate, 2 for elementary school, 3 for secondary school, and 4 for diploma and above) and wealth category (1 for poor, 2 for medium, and 3 for rich). All the other variables were continuous.

As the empirical models required the residuals to be normally distributed, the normal probability plot of residuals was compared with the ideal normal distribution plots to graphically inspect the level of divergence. The Shapiro–Wilk and Jarque–Bera statistical tests of normality were implemented on the model residuals. Both test the hypothesis that the residual is normally distributed and if the null is rejected, normality of residual is secured.

## 3 RESULTS

### 3.1 Socioeconomic characteristics and cropping patterns

There were significant differences (*α* = .05) in landholding size and livestock holdings among wealth categories in all the study areas (Table 1). The highest mean land and livestock holdings were observed at Welmel Tiqa, perhaps attributable to the recent transition from pastoral to mixed sedentary farming system in this area. Household head illiteracy, age, family size, livestock holding, landholding size, type of household head, and mean adult equivalent were significantly different (*α* = .05) among the three study sites.

**TABLE 1:** Socioeconomic characteristics of the respondents across three study sites in Ethiopia.

Variables	Mennissa (*n* = 84)				Welmel Tiqa (*n* = 98)				Agam Wuha (*n* = 83)			
	Rich	Medium	Poor	Total	High	Medium	Poor	Total	High	Medium	Poor	Total
Sex of HH head(%)												
Male‐headed	85.2a	88.9a	90a	88.1y	100a	86.1a	87.9a	90.8x	89.3a	77.8b	64.3c	77.1z
Female‐headed	14.8	11.1	10	11.9	0	13.9	12.1	9.2	10.7	22.2	35.7	22.9
Household head age	49.1 (2.3)a	43.6 (2.6)a	43.6 (2.5)a	45.4 (1.45)x	38.4 (1.8)a	37.9 (1.8)a	44.1 (2.8)a	40.1 (1.3)z	47.2 (2.26)a	37.9 (1.99)c	42.2 (2.3)b	42.5 (1.3)y
Household head education (%)												
Illiterate	18.5a	48.1a	40a	35.7z	27.6a	19.4a	27.3a	24.5y	39.3c	66.7b	85.7a	64x
Elementary	37	22.2	33.3	31	65.5	75	69.7	70.4	46.4	33.3	14.3	31.3
Secondary school	29.6	14.8	26.7	23.8	6.9	5.6	0	4.1	14.3	0	0	4.8
Diploma and above	14.8	14.8	0	9.5	0	0	3	1	0	0	0	0
Family size (in number)	7.6 (0.5)a	7.1 (0.5)a	6.5 (0.4)a	7.0 (0.3) y	9.9 (1.0)a	8.5 (0.7)a	8.9 (0.7)a	9.1 (0.5)x	6.1 (0.5)a	5.1 (0.4)a	4.7 (0.37)a	5.4 (0.24)z
Total adult equivalent (in AME)	6.4 (0.5)a	5.9 (0.4)a	5.3 (0.3)a	5.8 (0.2) y	7.7 (0.8)a	6.9 (0.6)a	7.2 (0.6)a	7.3 (0.4)x	5.5 (0.42)a	4.1 (0.31)b	3.9 (0.27)c	4.5 (0.21)z
Livestock holding (in TLU)	3.9 (0.5)a	2.6 (0.2)b	1.6 (0.1)c	2.7 (0.2)z	5.8 (0.8)a	3.3 (0.4)b	2.2 (0.4)c	3.7 (0.3)x	4.4 (0.52)a	2.9 (1.42)b	1.4 (0.26)c	2.9 (0.56)y
Total land holding size (ha)	1.7 (0.2)a	1.2 (0.2)b	0.6 (0.1)c	1.2 (0.1)y	2.9 (0.5)a	1.2 (0.1)b	0.8 (0.1)c	1.6 (0.2)x	1.3 (0.23)a	0.8 (0.10)b	0.7 (0.13)c	0.9 (0.10)z

*Note*: Results for continuous and categorical variables are presented in mean (SE) and percentage, respectively. Values followed by similar letters “a–c” across a row for a given study site (between wealth categories) and “x–z” between.

**FIGURE 3:**
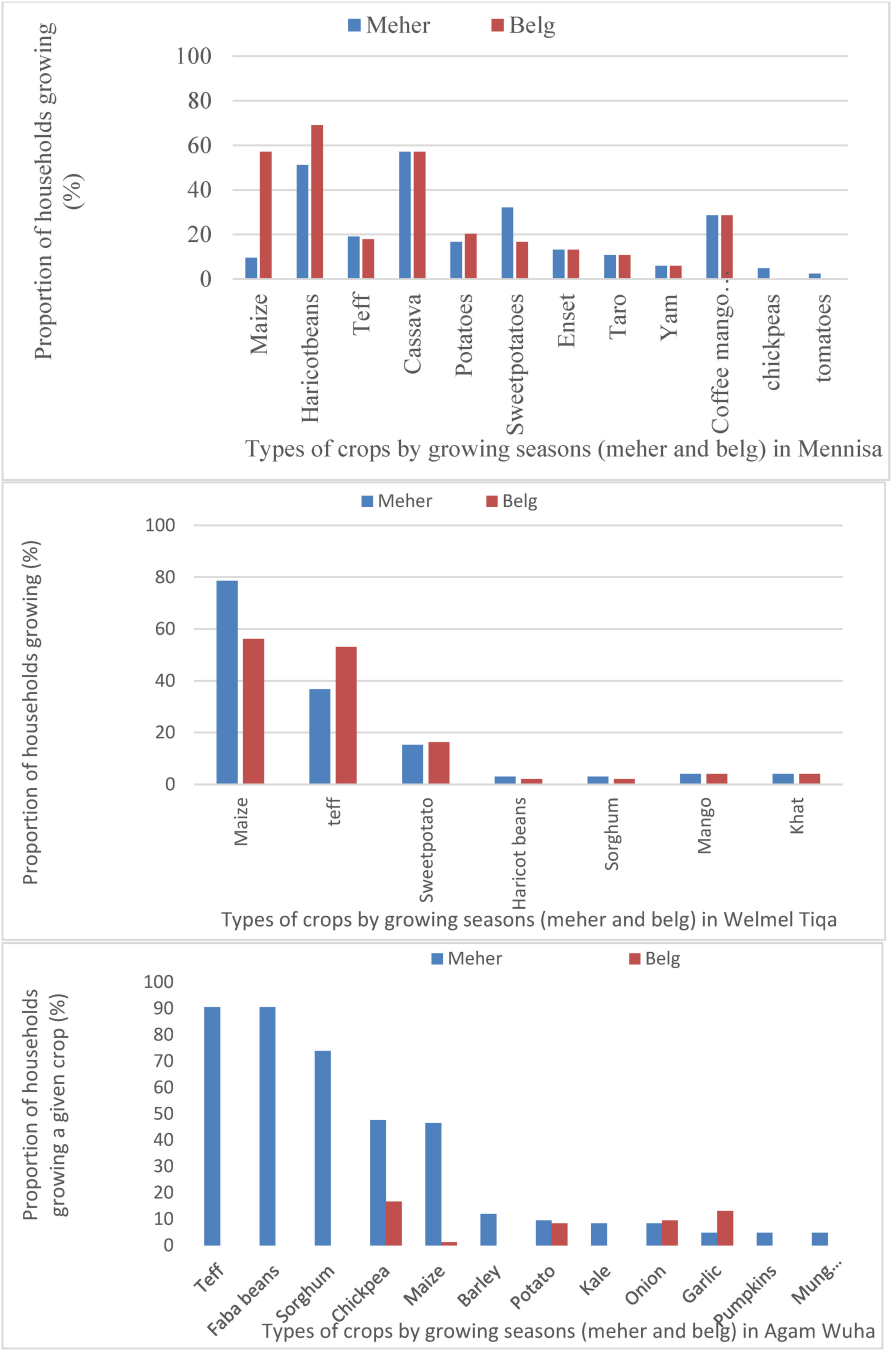
Major agricultural crops grown and proportion of respondents growing each crop at Belg and Meher seasons across the three study sites in Ethiopia.

The major types of crops and production systems were different among the three study sites (Figure 3). The highest crop diversity was observed at Mennisa where cereals (maize, teff, etc.), root crops (cassava, sweet potato, potato, taro, enset, and yam), legumes (haricot beans and chickpea), and fruits (mango, avocado, and banana) were all grown. The majority (83%, *n* = 84) of households in Mennisa grew six or more crops per year (three or more crops per rainfall season, Meher or Belg seasons) which are almost twice those grown in Welmel Tiqa and Agam Wuha areas. All respondents (100%, *n* = 84) in Mennissa, 89% of respondents (*n* = 98) in Welmel Tiqa, and 18% of respondents (*n* = 83) at Agam Wuha grew agricultural crops during both the short rainy season (Belg, February/March–April) and the long rainy season (Meher, June–September/October). The priority crops were cassava and haricot beans at Mennisa, maize and teff at Welmel Tiqa, and teff and beans at Agam Wuha areas (Figure 3). According to the focus group discussants in Mennissa, cassava is expanding at the expense of maize and other cereal crops, owing to its better productivity in suboptimal growing conditions, such as poor soil fertility and water stress, and importance as both sources of household consumption. Only a few respondents (14% in Welmel Tiqa and 8% in Mennisa) were engaged in irrigated farming.

### 3.2 Household income diversity and food expenditures

Smallholder farm households had a diversity of income sources, an average of 9 in Mennisa, 10 in Agam Wuha, and 11 in Welmel Tiqa (Table 2). The proportion of farm households receiving income from different sources and the contributions of each income source for the total annual household income were significantly different (*α* = .05) among the study sites. Being situated adjacent to the Harena forest (a remnant dense natural forest patch in Ethiopia), wild coffee was the most important source of income in Welmel Tiqa, contributing about 37% of the average total annual household income and engaging three quarters of smallholder farmers. In contrast, own produced agricultural crops were the most important sources of household income in Mennisa and Agam Wuha, accounting for 68.9% and 47.4% of the total annual income, respectively (Table 2). Although agricultural crops were the second most important income sources in Welmel Tiqa, there were a few recently arrived farm households from the nearby highlands who were not engaged in own crop production and depended on casual labor and small business income.

**TABLE 2:** Smallholder farmers' income sources and estimated share of each income source out of the total among the different wealth categories of the three study sites in Ethiopia.

Livelihood strategies as	Mennissa (*n* = 84)				Welmel Tiqa (*n* = 98)				Agam Wuha (*n* = 83)			
	Rich (%)	Medium (%)	Poor (%)	Total (%)	Rich (%)	Medium (%)	Poor (%)	Total (%)	Rich (%)	Medium (%)	Poor (%)	Total (%)
Own produced agricultural crops (%)												
Proportion of HHs engaging	100	100	100	100x	79.3a	61.1a	63.6a	67.3z	89.3a	92.6a	92.9a	91.6y
Share out of total income	66.0	64.4	60.7	68.9x	18.5	20.4	24.9	21.2z	49.6	48.7	44.0	47.4y
Own produced animal source foods and products (%)												
Proportion of HHs engaging	70.4a	63a	40a	57.1x	72.4a	30.6b	21.2c	39.8x	64.3a	55.6b	28.6c	49.4x
Share out of total income	15.4	14.3	8.8	12.9x	16.3	9.2	10.3	11.8x	17.2	18.3	8.1	14.5x
Harvested wild plants sources (mainly forest coffee) (%)												
Proportion of HHs engaging	0	0	0	0y	93.1a	77.8b	57.6c	75.5x	0	0	0	0y
Share out of total income	0	0	0	0y	34.3	42.0	34.1	36.3x	0	0	0	0y
Own produced/gathered goods/crafts (%)												
Proportion of HHs engaging	7.4a	11.1a	0a	6x	10.3a	8.3a	3.0a	7.1x	3.6a	11.1a	10.7a	8.4x
Share out of total income	1	3.7	0	1.8x	7.2	2.1	2.4	3.5x	2	4.4	4.3	3.5x
Casual labor and daily wages (%)												
Proportion of HHs engaging	3.7b	3.7b	36.7a	15.5z	17.2a	30.6a	18.2a	22.4y	10.7c	44.4b	50a	34.9x
Share out of total income	0.3	0.5	12.2	5.38x	3.8	7.6	5.8	5.8x	2.4	12.7	13.2	9.4x
Medium and small business such as petty trades (%)												
Proportion of HHs engaging	7.4a	7.4a	20a	11.9z	51.7a	22.2b	6.1c	25.5y	32.1a	22.2a	46.4a	33.7x
Share out of total income	1	1.4	8.5	3.8z	16.1	5.6	6.1	8y	14.4	11.7	23.9	16.8x
Permanent/contractual employment (%)												
Proportion of HHs engaging	18.5a	14.8a	6.7a	13.1x	3.4a	0a	0a	1z	3.6a	0a	3.6a	2.4y
Share out of total income	8.1	5	1.3	4.5x	0.1	0	0	0z	3.6	0.0	0.8	1.5y
Remittance (%)												
Proportion of HHs engaging (%)	3.7a	11.1a	3.3a	6.0x	0a	5.6a	3a	3.1x	7.1a	0a	0a	2.4x
Share out of total income	0.6	4.14	1.11	2.3x	0	2.16	1.56	1.32x	1.1	0	0	0.4x
Public cash or kind transfer												
Proportion of HHs engaging	3.7a	0a	6.7a	3.6z	13.8a	22.2a	33.3a	23.5x	0a	3.7a	10.7a	4.8y
Share out of total income	0.9	0	2.8	1.5y	1.6	7.6	11.8	7.2x	0	1.8	0.95	0.92z
Renting farmland and equipment (%)												
Proportion of HHs engaging	29.6a	18.5a	16.7a	21.4x	24.1a	0b	0b	7.1z	7.1a	11.1a	17.9a	12y
Share out of total income	6.7	6.7	4.7	6.38x	4.0	0	0	1.1z	2.4	2.2	4.6	3.1y
Other income sources (%)												
Proportion of HHs engaging	0	0	0	0x	10.3a	5.6a	3.0a	6.1x	17.9a	0b	0b	6.0x
Share out of total income	0	0	0	0x	1.2	4.6	3.0	3.1x	7.3	0	0	2.5x

*Note*: Results for continuous and categorical variables are presented in mean (SE) and percentage, respectively. Values followed by similar letters “a–c” across a row for a given study site (between wealth categories) and “x–z” between study sites are not significantly different.

The proportion of households receiving income from different sources and the contributions of each income source toward total annual income varied significantly (*α* = .05) by wealth category in the study sites (Table 2). Casual labor was the second most important source of income for the poor, while livestock was second most important income source for the rich at Mennisa. High proportion of the households in the Mennisa (57.1%) and some households in Welmel Tiqa (39.8%) were earning income by selling animal products. Medium‐scale businesses, such as grinding mills and rural shops, were more important for the rich, while public cash transfer from the Safety Net program, including paid labor for community services such as maintenance of rural roads, was important for the poor in Welmel Tiqa.

**FIGURE 4:**
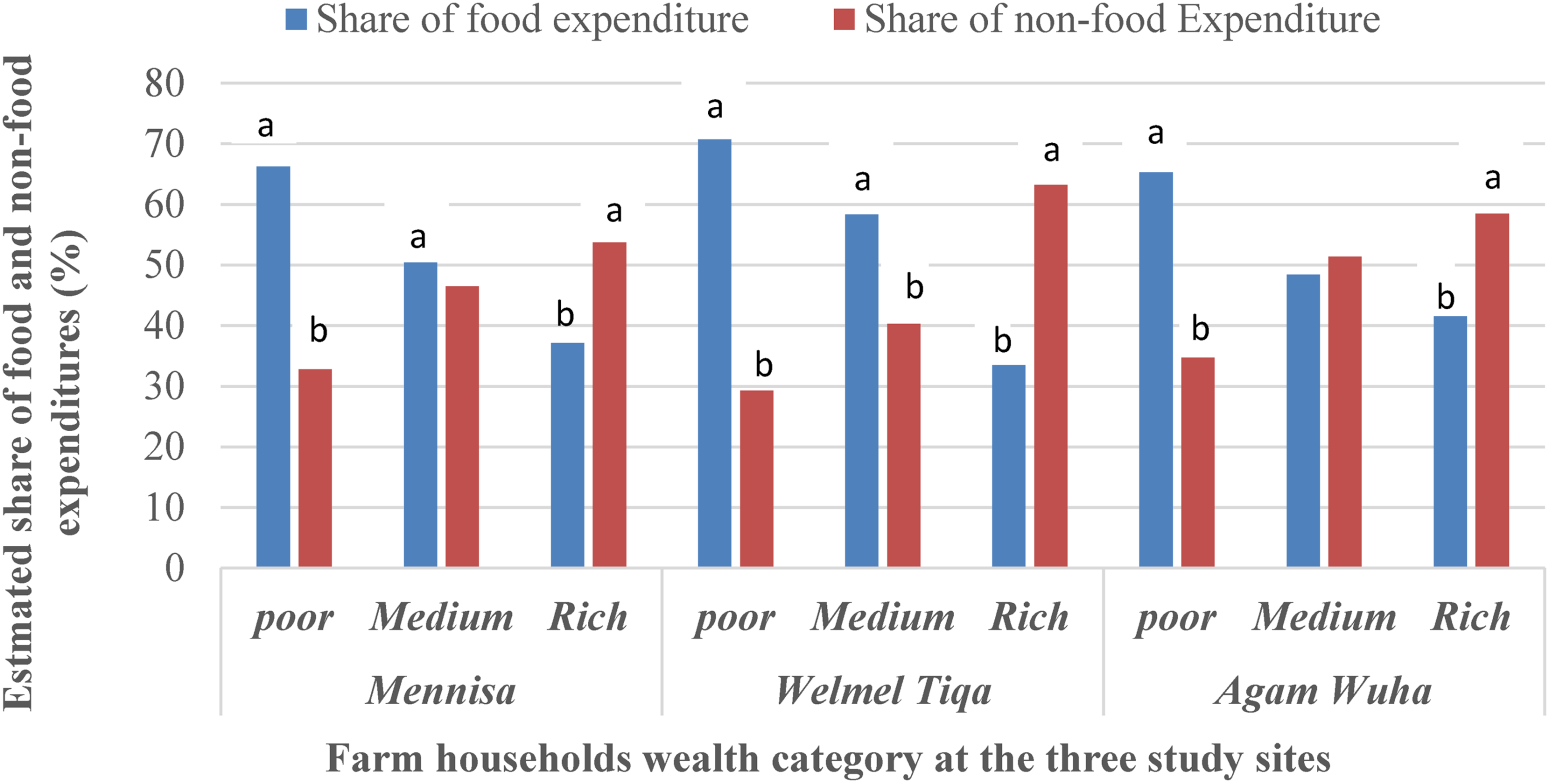
Share of food and non‐food expenditures out of the total annual household incomes at the three study sites of Ethiopia. Values followed by dissimilar letters of a–b in each wealth category and a given study site are meant for significantly (*α* = .05) different and vice versa. When the sum of the food and no‐food expenditures was not 100%, the difference is accounted as saving.

The shares of the food and non‐food expenditures were significantly different among the poor and rich wealth categories in the study sites (Figure 4). The share of food expenditures was significantly (*α* = .05) higher than that of the non‐food expenditures for the poor. However, the share of the non‐food expenditures was significantly higher than that of the food expenditures for the rich across all the three study sites.

### 3.3 FVS and household nutrition profiles

**FIGURE 5:**
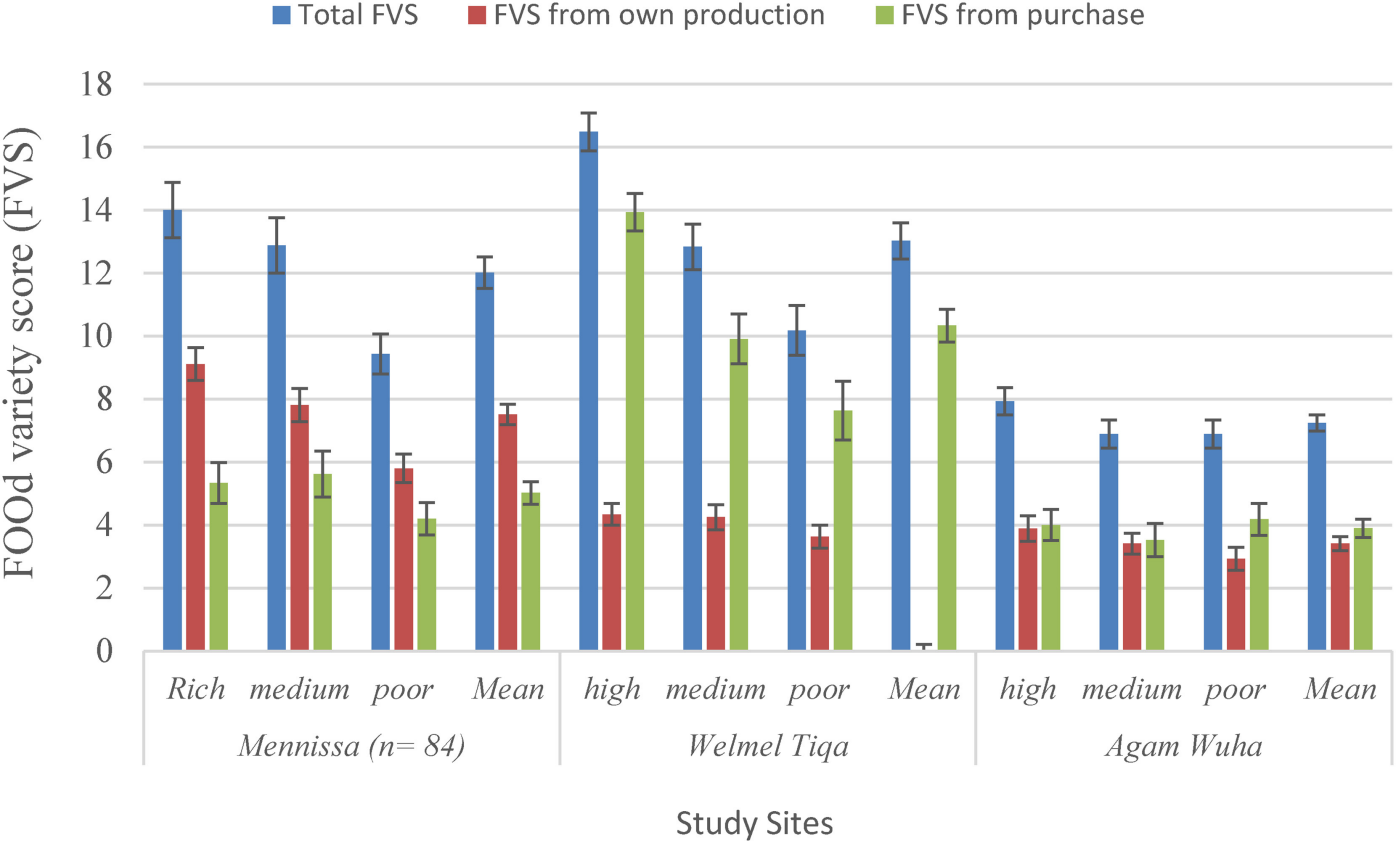
Food variety scores from own production and purchase among different wealth categories at the three study sites in Ethiopia.

There were differences in FVSs among study sites and wealth categories (Figure 5). The mean total FVS at Welmel Tiqa was twice that of the mean total FVS at Agam Wuha. Moreover, the total food FVSs were significantly (*p* < .05) different by wealth categories in Mennisa and Welmel Tiqa, but not in Agam Wuha (Figure 5). Hence, the total FVSs of the rich were 32.64% and 38.2% greater than those of the poor at Mennisa and Welmel Tiqa areas, respectively. The contributions of own production and purchase to the total FVSs also varied among wealth categories in the three study sites (Figure 5). Accordingly, FVS from own production was higher than that from purchase at Mennisa, while the latter was higher at Agam Wuha and Welmel Tiqa (Figure 5).

There were significant differences in nutrition profiles of farm households, both by study sites and wealth categories within each site (Table 3). Although the apparent intake of energy and other nutrients (protein, zinc, iron, magnesium, and Vitamins: B_1_, B_3_, and B_6_) were above the EARs at the population level, there were still significant proportions of the farm households that were below the EARs at all study sites. The result of this study revealed that Vitamin A, calcium, Vitamin B_12_, and Vitamin D were apparently below EAR cutoffs (at higher risk for inadequate apparent intake) at the population level, in all the three study sites. However, there were spatial differences in terms of adequacy of some micronutrients. The apparent intakes of calcium were below the EAR at population level at Welmel Tiqa and Agam Wuha, but above the EAR at population level at Mennisa. Folic acid and Vitamin B_2_ were taken below the EAR only at Welmel Tiqa. Vitamin E was taken below the EAR only at Agam Wuha. The differences in the apparent intake of micronutrients among the study sites could be attributed to the farming systems, food markets, and culinary food habits.

**TABLE 3:** Average apparent energy and nutrient intakes and prevalence of households at higher risk of inadequate apparent intake (% below EAR for AME) in the three study sites of Ethiopia.

Type of nutrient	Wealth category	Mennissa/Root crops‐based farming (84)		Welmel Tiqa/Maize‐based semi‐pastoral farming (*n* = 98)		Agam Wuha/Teff‐based cereal farming systems (*n* = 83)	
		Median (IQR)	Households with risk of Inadequacy (%)	Median (IQR)	Households with risk of Inadequacy (%)	Median (IQR)	Households with risk of Inadequacy (%)
Energy (kcal)	Rich	3608 (1969, 4449)a	37	4071 (2171, 4994)a	31	3059 (1958, 3765)a	35.7
	Medium	2668 (2042, 4402)b	48.1	3190 (1794, 4253)b	41.7	2830 (1948, 4523)a	44.4
	Poor	1978 (1266, 3094)c	63.3	2607 (2212, 4178)c	40.6	2901 (1732, 3732)a	35.7
	Total	2519 (1820, 3920)	50	3146 (1983, 4431)	38.1	2906 (1949, 3796)	38.6
	EAR	2488.4					
Protein (g)	Rich	93 (58, 120)a	7.4	84 (47, 126)a	17.2	68.9 (56.5, 93.2)a	14.3
	Medium	72 (48, 87)b	7.4	67 (37, 125)a	36.1	64.3 (43.1, 116.6)a	25.9
	Poor	44 (27, 54)c	50	69 (38, 113)a	25	59.5 (34.6, 86.5)a	25
	Total	60 (44, 98)	22.6	74 (41, 121)	26.8	67.6 (47.6, 94)	21.7
	EAR	43.2					
Zinc (mg)	Rich	18.7 (10.6, 22)a	18.5	25 (12.6, 32)a	10.3	20.7 (14.3, 27.6)a	7.1
	Medium	13.9 (9.4, 19.9)b	22.2	17.3 (9.1, 30)a	27.8	18.9 (12.8, 32.0)a	11.1
	Poor	7.9 (4.57, 11.5)c	66.7	17.8 (11.1, 33)a	21.9	18.3 (11.4, 24.1)a	17.9
	Total	11.7 (7.9, 19.0)	36.9	18.9 (10.8, 32)	20.6	20.0 (13.2, 27.2)	12
	EAR	9.4					
Iron (mg)	Rich	66 (30, 82)a	0	93 (51, 133)a	0	78.1 (59.7, 103.6)a	0
	Medium	45 (33, 66)b	0	81 (38, 121)a	0	68.0 (56.5, 113.2)a	0
	Poor	22 (17.1, 36)c	3.3	69 (37, 122)a	6.2	64.7 (42.8, 96.9)a	0
	Total	38 (23, 66)	1.2	78 (43, 126)	2.1	70.7 (54.2, 99.0)	0
	EAR	6					
Vitamin A (μg RAE)	Rich	127 (40, 259)a	100	37 (17.1, 67)a	100	183 (58.1, 246)a	89.3
	Medium	115 (46, 190)b	100	56 (25, 127)a	100	179 (72.5, 262)a	100
	Poor	48 (26, 113)c	100	48 (13.8, 83)a	100	160.2 (45.9, 347)a	92.9
	Total	80 (34, 178)	100	49 (17.0, 86)	100	179 (61.1, 285)	94
	EAR	625					
Calcium (mg)	Rich	847 (639, 1419)a	40.7	747 (410, 1000)a	62.1	665 (455, 907)a	71.4
	Medium	775 (532, 1014)b	55.6	511 (325, 800)b	77.8	667 (418, 1190)a	59.3
	Poor	440 (269, 650)c	83.3	402 (229, 622)c	87.5	582 (359, 966)a	71.4
	Total	687 (433, 1089)	60.7	523 (320, 797)	76.3	628 (418, 1002)	67.5
	EAR	800					
Folic acid (μg)	Rich	885 (600, 1255)a	0	376 (271, 507)a	37.9	648 (484, 959)a	7.1
	Medium	729 (510, 1003)b	0	261 (184, 396)c	61.1	579 (374, 1152)a	18.5
	Poor	445 (304, 654)c	30	265 (142, 374)b	65.6	632 (459, 823)a	14.3
	Total	628 (433, 980)	10.7	302 (189, 437)	55.7	604 (457, 951)	13.3
	EAR	320					
Vitamin C (mg)	Rich	174 (100, 232)a	11.1	60 (37, 97)a	69	40.7 (25.6, 62.8)a	82.1
	Medium	147 (85, 187)a	14.8	56 (34, 90)a	66.7	42.0 (28.8, 74.0)a	74.1
	Poor	151 (89, 230)a	16.7	45 (31, 70)a	78.1	52.5 (29.7, 76.5)a	71.4
	Total	156 (90, 218)	14.3	53 (35, 80)	71.1	42.1 (28.9, 74.0)	75.9
	EAR	75					
Vitamin B_12_ (μg)	Rich	0.000 (0.000, 0.056)b	100	0.231 (0.024, 0.535)a	100	0.50 (0.000, 1.34)a	92.9
	Medium	0.000 (0.000, 0.069)a	96.3	0.108 (0.000, 0.325)b	100	0.389 (0.000, 1.04)a	92.6
	Poor	0.000 (0.000, 0.000)c	100	0.000 (0.000, 0.051)c	100	0.106 (0.000, 0.630)a	100
	Total	0.000 (0.000, 0.000)	98.8	0.048 (0.000, 0.299)	100	0.331 (0.000, 0.948)	95.2
	EAR	2					
Vitamin D (μg)	Rich	0.000 (0.000, 0.000)a	100	0.203 (0.000, 0.325)a	100	0.000 (0.000, 0.000)a	100
	Medium	0.000 (0.000, 0.122)a	100	0.000 (0.000, 0.191)b	100	0.000 (0.000, 0.000)a	100
	Poor	0.000 (0.000, 0.000)a	100	0.000 (0.000, 0.057)c	100	0.000 (0.000, 0.000)a	100
	Total	0.000 (0.000, 0.000)	100	0.000 (0.000, 0.199)	100	0.000 (0.000, 0.000)	100
	EAR	10					
Vitamin B_1_ (mg)	Rich	2.91 (1.75, 3.64)a	0.0	3.07 (1.56, 3.86)a	6.9	2.464 (1.593, 3.120)a	3.6
	Medium	2.36 (1.57, 3.12)b	3.7	1.99 (1.18, 3.31)a	11.1	2.013 (1.590, 3.671)a	3.7
	Poor	1.42 (1.01, 2.09)c	23.3	1.93 (1.34, 3.09)a	9.4	2.143 (1.457, 2.985)a	14.3
	Total	2.03 (1.42, 3.10)	9.5	2.07 (1.45, 3.61)	9.3	2.214 (1.590, 3.190)	7.2
	EAR	1					
Vitamin B_2_ (mg)	Rich	1.55 (0.95, 2.07)a	37.0	1.32 (0.76, 1.69)a	37.9	1.390 (0.975, 1.820)a	32.1
	Medium	1.19 (0.87, 1.57)b	40.7	0.96 (0.52, 1.42)b	63.9	1.346 (0.940, 2.397)a	37
	Poor	0.84 (0.48, 1.21)c	73.3	0.76 (0.57, 1.21)c	71.9	1.351 (0.951, 2.100)a	32.1
	Total	1.10 (0.83, 1.56)	51.2	0.97 (0.58, 1.45)	58.8	1.351 (0.955, 2.100)	33.7
	EAR	1.1					
Vitamin B_3_ (mg)	Rich	22.2 (14.4, 28.8)a	11.1	22.8 (13.0, 32.6)a	20.7	15.60 (11.61, 22.80)a	25
	Medium	18.2 (13.6, 27.2)b	11.1	20.8 (12.8, 26.1)a	19.4	14.60 (11.00, 27.17)a	29.6
	Poor	13.4 (8.0, 21.6)c	40.0	20.4 (15.1, 34.1)a	15.6	14.67 (8.21, 21.40)a	35.7
	Total	17.4 (12.6, 26.8)	21.4	21.6 (13.2, 30.9)	18.6	14.85 (8.21, 21.40)	30.1
	EAR	12					
Vitamin B_6_ (mg)	Rich	4.48 (2.64, 6.00)a	0.0	3.54 (1.88, 5.07)a	3.4	3.61 (2.34, 4.37)a	0
	Medium	3.49 (2.51, 5.37)b	0.0	3.51 (1.82, 4.32)a	8.3	3.13 (2.50, 5.99)a	3.7
	Poor	2.58 (1.59, 3.75)c	13.3	2.88 (2.19, 4.47)a	6.3	3.55 (2.38, 4.93)a	10.7
	Total	3.20 (2.33, 5.16)	4.8	3.21 (2.03, 4.48)	6.2	3.56 (2.46, 4.96)	4.8
	EAR	1.1					
Vitamin E (mg)	Rich	14.9 (7.4, 24.0)a	44.4	20.9 (13.6, 34.2)a	20.7	4.53 (3.27, 6.65)a	89.3
	Medium	15.7 (12.1, 26.2)a	22.2	16.0 (11.9, 25.6)a	25.0	4.28 (2.82, 7.26)a	85.2
	Poor	15.4 (5.34, 22.0)a	36.7	15.4 (9.6, 22.0)a	37.5	4.62 (2.92, 8.42)a	89.3
	Total	15.4 (9.9, 22.8)	34.5	16.6 (11.6, 26.4)	27.8	4.53 (3.07, 7.26)	88
	EAR	12					
Magnesium (mg)	Rich	961 (573, 1188)a	0.0	1188 (603, 1506)a	6.9	1152 (710, 1389)a	3.6
	Medium	776 (508, 1082)b	3.7	674 (452, 1367)a	11.1	1017 (706, 1389)a	0
	Poor	428 (325, 659)c	30.0	811 (533, 1300)a	9.4	1032 (668, 1496)a	10.7
	Total	641 (450, 1028)	11.9	859 (551, 1465)	9.3	1076 (706, 1542)	4.8
	EAR	350					

There were significant differences in the apparent intake of nutrients among wealth categories at Mennisa and Welmel Tiqa, but not at Agam Wuha (Table 3). In Mennisa, the apparent intake of all nutrients, except Vitamin C, Vitamin D, and Vitamin E, was significantly (*p* < .05) different among the wealth categories. On the other hand, the apparent intake of Energy, Calcium, Folic acid, Vitamin B_2_, and Vitamin B_12_ was significantly different among wealth categories in Welmel Tiqa. The result of this study revealed that the effect of wealth category on apparent intake of nutrients is context specific, as it is heavily influenced by the availability of the different food items from own production and on the market, and local culinary systems.

### 3.4 Determinants for farm household nutrition profiles

The number of adequately consumed nutrients by farm households, also considered as the number of nutrients above EAR cutoff levels, was associated with family size, age of household heads, livestock holdings, crop production diversity, income diversity, wealth categories, FVS, and irrigation use (Table 4). Positive associations were observed with crop production diversity in Belg season, income diversity, and FVSs (Table 4). A unit increase in the number of crop types grown in Belg season could lead to an increase in the number of nutrients adequately consumed at a household level by 0.8 in Agam Wuha and 1.0 in Welmel Tiqa. An increase in family size by one member reduced the number of nutrients adequately consumed at a household level by 0.4 in Mennisa and 0.2 in Welmel Tiqa. Likewise, an increase in the number of food items (FVS) consumed significantly increased the number of nutrients adequately consumed at a household level by 0.2 in Mennisa and 0.3 in Welmel Tiqa and Agam Wuha. An increase in the number of household's income sources by 1 increased the number of nutrients adequately consumed at a household level by 0.7 in Mennisa. Unexpectedly, livestock holding units (TLU), crop production diversity in Meher season, and irrigation use were significantly and negatively associated with nutrition adequacy of households at Welmel Tiqa (Table 4). The relationship between livestock ownership and nutrition adequacy should be interpreted cautiously. In general, it is known that many rural households keep livestock as a store of wealth and considered as permanent assets, instead of a readily available food source. Keeping all other determinants of nutrition adequacy the same, living in the medium and poor households reduced the number of nutrients adequately consumed by 1 and 1.8 at the whole study population level, implying that transforming from a lower to higher wealth category would improve the nutrition profile of farm households.

**TABLE 4:** Determinants for household nutrition security at Mennissa, Welmel Tiqa, and Agam Wuha areas of Ethiopia.

Variables	Entire sample	Agam Wuha	Welmel Tiqa	Mennisa
Landholding size	0.175 (0.152)	0.165 (0.467)	0.238 (0.176)	0.218 (0.340)
Family size	−0.229*** (0.0560)	−0.108 (0.176)	−0.193*** (0.0664)	−0.367*** (0.135)
Household head sex: female	−0.337 (0.484)	0.00894 (0.840)	0.623 (0.990)	−0.766 (0.878)
Age of household head	−0.0277* (0.0147)	−0.0300 (0.0296)	−0.0409 (0.0252)	−0.0358 (0.0253)
Household head education: elementary	−0.539 (0.375)	−0.945 (0.638)	−0.722 (0.692)	0.216 (0.708)
Household head education: secondary	−0.943 (0.602)	−1.071 (1.633)	−0.780 (1.634)	−0.0294 (0.758)
Household head education: diploma/above	−0.813 (0.929)		−1.415 (2.581)	−0.635 (1.028)
Livestock holding (TLU)	−0.145* (0.0822)	−0.108 (0.214)	−0.279** (0.112)	0.0182 (0.176)
Crop production diversity (*Belg* season)	0.185* (0.110)	0.808* (0.450)	1.068** (0.514)	0.289 (0.240)
Crop production diversity (*Meher* season)	0.192 (0.118)	0.317 (0.230)	−0.832* (0.487)	0.325 (0.203)
Status on irrigation use (yes/no)	−0.536 (0.420)	−0.661 (0.806)	−2.294*** (0.811)	0.419 (1.009)
Income diversity score	−0.0278 (0.159)	−0.228 (0.399)	−0.127 (0.229)	0.650* (0.383)
Total annual income	−4.50e‐06 (3.50e‐06)	2.10e‐05 (2.61e‐05)	−2.52e‐06 (4.50e‐06)	−1.66e‐05 (1.53e‐05)
Food expenditure % out of total income	0.00202 (0.00735)	0.0194 (0.0129)	0.00105 (0.0134)	4.51e‐05 (0.0129)
Food variety score	0.215*** (0.0411)	0.342*** (0.126)	0.307*** (0.0633)	0.162** (0.0745)
Wealth categories: medium	−0.968** (0.487)	−0.514 (1.151)	−1.609 (0.970)	0.382 (0.805)
Wealth categories: poor	−1.759*** (0.559)	−0.776 (1.041)	−1.065 (1.237)	−1.391 (1.015)
Intercept term	9.975*** (1.152)	6.779*** (2.411)	10.17*** (2.039)	8.053*** (2.700)
Sigma	2.491*** (0.112)	2.379*** (0.185)	2.236*** (0.174)	2.231*** (0.173)
Observations	252	83	86	83

*Note*: SEs in parentheses.

*** *p* < .01;

** *p* < .05;

* *p* < .1.

## 4 DISCUSSION

Owing to the differences in agro‐ecological conditions and farming systems among the three study sites, farm household's income diversity and the role of livelihood strategies to the total household income were different among study sites. Smallholder farmers try to sustain and improve their existence through diversifying their livelihood strategies (Loison, 2015). The contributions of wild plants to the food and incomes of farm households vary by setting. Forest coffee is the most important wild plant with the largest income contributor among the 11 livelihood strategies in Welmel Tiqa (Tadesse & Mbongo, 2004). Hence, the largest proportions of the household incomes were obtained from the sale of coffee and livestock, resulting in sourcing much of their food from purchase than from own production in Welmel Tiqa. In the root crops‐based and cereals‐based farming systems of Mennisa and Agam Wuha areas, smallholder farmers earn much of their incomes from their own agricultural production. The higher contribution of own production for the total FVSs in all households, regardless of the wealth class in Mennisa, was likely driven by higher crop production diversity. In Agam Wuha, where the crop production diversity was low, the total FVS was painfully low and the contribution of own production for the total FVS was slightly less than that purchased from niche market. These results imply that the nutrition security status of the farm households could be intrinsically and contextually interlinked to both what they produce in their own farm and how much they earn from different livelihood strategies. Based on a study in Kenya and Malawi, Sibhatu et al. (2015) revealed that increasing on‐farm diversity can have a positive effect to improve the food and dietary diversity of smallholder farm households, although it should not be considered the only means to ensure nutrition security.

Poor households, which have consistently lower land and livestock holdings across study sites, had significantly higher proportion of expenditures spent on food than on non‐food items. This is in line with a previous study elsewhere in Africa that revealed food expenditures range between 60% and 80% of total household income for low‐income households (Baiphethi & Jacobs, 2009). Hence, household food demand might not be satisfied by the production from small landholdings or just a few livestock thus forcing them to opt for other income sources, and be able to rely more on food purchases. In all the study sites, the poor were much more dependent on other income sources, including the productive Safety Net program support (typically food for work programs), casual labor, and small businesses. They made it more probable to have low FVSs and hence, higher nutrition insecurity. This implies that nutrition‐sensitive agricultural interventions should be pro‐poor, emphasizing enhanced opportunities for income earning along the entire agricultural value chains as well as access to seeds of diversified nutritious crops and nutritional knowledge.

Although there were differences in the household dietary intake for many of the nutrients among study sites and wealth categories, the four micronutrients (Vitamins A, B_12_, D, and calcium) were consistently least consumed, indicating a high risk of hidden hunger in the study areas. According to National Nutrition Program I launched in 2009, the estimated prevalence of Vitamin A deficiency (VAD) was 61% among children under 5 years of age (Government of the Federal Democratic Republic of Ethiopia (GFDRE), 2013). This is higher than the level for similar age groups in SSA, which was estimated at 48% (Low et al., 2017). Although more than half of the farm households were observed to have apparent intake of many of the nutrients above EAR levels, there are still many households that have apparent intakes below the EAR. Government estimates indicate that close to 50,000 Ethiopian children die each year from Vitamin A, iron, and folic acid deficiencies (GFDRE, 2011). Some studies in Ethiopia reported the intake of poor diet quality by different segments of the population and its association with different forms of undernutrition problems (Abdulahi et al., 2017; Getaneh et al., 2019; Kuche et al., 2020). Food‐based approaches are one of the more long‐term, sustainable strategies for reducing the micronutrient deficiencies in developing countries such as Ethiopia. In this context, consumption of foods that are naturally rich in micronutrients or enriched through industrial fortification or breeding should be promoted.

The number of nutrients adequately taken by a given farm household was determined by crop production diversity, income diversity, FVSs, age of household head, household wealth status, family size, livestock holding, and irrigation use. The first two determinants had positive associations, implying that enhancing household incomes and diversifying the crops produced would enhance FVSs and nutrition security. Therefore, agricultural value chains development and improving employment opportunities of farm households can enhance the nutrition security indirectly by improving household incomes. This may need to be aptly integrated with improved market development and concurrent nutrition education efforts. Smallholder farmers in Ethiopia and other developing countries also purchase foods that they access from niche and regular markets and consume at their homes during the lean season. This is especially the case for specialized farmers or cash crop producers, who may even have such access during the harvest season (Amede et al., 2008; Ayenew et al., 2018; Hirvonen et al., 2017; Jones, 2017; Jones et al., 2014; Koppmair et al., 2017; Romeo et al., 2016; Sibhatu & Qaim, 2017, 2018a, 2018b; Sibhatu et al., 2015; Zanello et al., 2018). Increasing crop diversification in smallholder farming by introducing additional crop and livestock species is crucial for improving the diet quality and nutritional status of these household members, as the majority of the smallholder farm households are consuming what they produce at household level in developing countries (Sibhatu & Qaim, 2017). It is also one of the strategies for protecting or decreasing weather and market‐related shocks, and thus also contributing for sustainable agriculture and land management (Romeo et al., 2016). Moreover, increasing the off‐farm income through market access also increased with diet diversity, even in some situations its contribution to diet quality was better than production diversity (Sibhatu & Qaim, 2017; Sibhatu et al., 2015). Therefore, identifying the contribution of production diversity, market access, and supporting households for establishing off‐farm income at different contexts will enhance the quality of diet at the farm households. For instance, income is more important in areas where farm households might not be able to increase the diversity of crops to be produced due to poor growing conditions. Regardless of these conditions, agriculture should be nutrition‐sensitive to produce nutritious food and increase availability in the locality.

Food and Agriculture Organization (FAO) also advocates comprehensive food‐based approaches encompassing food production, dietary diversification, and food fortification as sustainable strategies for improving the micronutrient status of populations and they should be implemented through practical and science‐based evidences (Thompson & Amoroso, 2010). Despite lots of efforts, the number of undernourished people remains high and even increased from 785.4 million to 821.6 million between 2015 and 2018 (FAO, IFAD, UNICEF, 2019). The government of Ethiopia has substantially invested a lot to reduce undernutrition problem and registered remarkable progress in reducing undernutrition from 41% to 21% for underweight, and 58% to 37% for stunting among children under 5 years of age (CSA, 2016; EPHI & ICF, 2019). However, much remains to be done to lower the prevalence of maternal and child malnutrition and micronutrient deficiencies (CSA, 2017; Ethiopian Public Health Institute, 2016). One example is the encouraging effort to address Vitamin A deficiency through proper introduction and disseminations of biofortified orange‐flesh sweet potato (OFSP) varieties across the SSA (Low et al., 2017, 2020). Moreover, OFSP‐based Vitamin A‐rich recipes are being developed for Ethiopia that integrates the crop into traditional dishes. Yet, this should be strengthened through enhanced nutrition educations and cooking demonstrations in many sites.

Although the existing number of irrigation using households in Welmel Tiqa was very low, the negative association between irrigation use and nutrient adequacy might be due to the fact that most farmers produce commercial and high‐value crops like khat, green maize, and sweet potato for sale, not for household consumption. The income generated from the sale of these irrigated crops was not necessarily directly translated into improved consumption and nutrition security. In these male‐dominated communities, the distribution of income within the household is highly asymmetric and women have limited control over the income that comes from the sale of those high‐value irrigated crops. It is very likely that irrigation using farmers may face food and nutrition insecurity due to the disproportionate use of irrigation for high‐value crops and ineffective allocation of income for enhancing household nutritional outcomes. The recent emphasis on increasing investments in irrigated agriculture developments should aptly consider the integrated gender‐based and nutrition‐sensitive interventions encompassing introduction of nutrient rich and/or biofortified crops, nutrition education campaigns, and cooking demonstrations to adapt new recipes with local culinary systems. This study has much strength, which includes that our study is the first one in Ethiopia that considered the 7‐day family food acquisition data to estimate energy and nutrient intake, and determine the prevalence of their adequacies at household level; and also identified determinants for the number of adequate nutrients at household levels in different farming systems of rural Ethiopia. Despite these, considering equity in intrahousehold food distribution among family members and taking physiologically nutrient needy mothers (pregnant and lactating) as non‐pregnant‐non‐lactating mother were the limitations of this study.

## 5 CONCLUSION

Smallholder farmers strive to improve their food security situation through practicing a diversity of livelihood strategies that supplement crop and livestock production. There were significant differences in crop production diversity, income diversity, and overall income of farm households among the three study sites. Beyond income diversity, the contribution of each income source for the total household incomes varied between the study sites and among the wealth categories in each study site. The strong dependence of the smallholder farmers on the wild coffee at Welmel Tiqa revealed that wild plants can still be crucial in the livelihoods and hence, food systems of the local community. The proportion of expenditures on food was significantly higher than that on non‐food items for the poor, while the non‐food expenditures were significantly higher than those for food among the rich households, irrespective of differences in the farming system practiced. Although there were observed differences in households' nutrition profiles among wealth categories, Vitamin A, B_12_, and D, and calcium intake levels were apparently much below EAR values and hence, local communities are at higher risks of hidden hunger that should be addressed through agro‐food interventions. Household nutrition security was negatively associated with family size, age of household head, livestock holdings, irrigation use, and wealth categories, and positively associated with crop production diversity, income diversity, and FVS. Although both crop production diversity and income diversity positively influenced the number of adequately consumed nutrients by a farm household, they could not guarantee meeting all micronutrient needs on average. It can be concluded that the FVS can be used as an indicator for monitoring nutrient adequacy at farm households in the study sites. Yet, this needs further validation for its applicability to find the exact score that captures the real nutrition security situation, including micronutrient intake, in each rural setting of Ethiopia. The negative association between irrigation use and number of adequately consumed nutrients could be due to the current practice of prioritizing high‐value commercial crops such as khat and perishable vegetables. It is very likely that irrigation using farmers may face food and nutrition insecurity due to the disproportionate use of irrigation for high‐value crops and ineffective allocation of income for enhancing household nutritional outcomes. The recent growing investments in irrigated agriculture value chain development should aptly consider gender‐transformative and nutrition‐sensitive interventions encompassing introduction of nutrient‐rich and biofortified crops, nutrition education campaigns, and cooking demonstrations to adapt new recipes with local culinary systems. Therefore, it is recommended that context‐based household nutrition profile assessments should be undertaken for effective planning and monitoring of nutrition‐sensitive irrigated agriculture development interventions.

## AUTHOR CONTRIBUTIONS

**Beruk Berhanu Desalegn:** Conceptualization (lead); data curation (equal); formal analysis (equal); investigation (equal); methodology (lead); resources (supporting); software (lead); supervision (lead); validation (equal); visualization (equal); writing – original draft (lead); writing – review and editing (equal). **Birhanu Biazin:** Conceptualization (lead); data curation (equal); formal analysis (equal); funding acquisition (equal); investigation (equal); methodology (lead); project administration (lead); resources (equal); software (equal); supervision (lead); validation (equal); visualization (equal); writing – original draft (equal); writing – review and editing (equal). **Tilahun Amede:** Conceptualization (equal); data curation (equal); funding acquisition (lead); investigation (equal); methodology (equal); project administration (lead); resources (lead); supervision (equal); writing – review and editing (equal). **Jan Low:** Conceptualization (supporting); data curation (equal); methodology (equal); supervision (supporting); writing – review and editing (equal).

## CONFLICT OF INTEREST STATEMENT

The authors declare that they have no competing interests.

## Data Availability

The datasets analyzed during the current study are available from the corresponding author upon reasonable request.
